# Dehydroxylated Polyvinyl Alcohol Separator Enables Fast Kinetics in Zinc‐Metal Batteries

**DOI:** 10.1002/smll.202410758

**Published:** 2025-01-26

**Authors:** Yao Qin, Fuhua Yang, Jodie A. Yuwono, Alberto Varzi

**Affiliations:** ^1^ Helmholtz Institute Ulm (HIU) Helmholtzstrasse 11 89081 Ulm Germany; ^2^ Karlsruhe Institute of Technology (KIT) P.O. Box 3640 76021 Karlsruhe Germany; ^3^ School of Chemical Engineering The University of Adelaide Adelaide SA 5005 Australia

**Keywords:** dehydroxylation, PVA, separators, zinc dendrites, zinc‐metal batteries

## Abstract

Separators are critical components of zinc‐metal batteries (ZMBs). Despite their high ionic conductivity and excellent electrolyte retention, the widely used glass fiber (GF) membranes suffer from poor mechanical stability and cannot suppress dendrite growth, leading to rapid battery failure. Contrarily, polymer‐based separators offer superior mechanical strength and facilitate more homogeneous zinc (Zn) deposition. However, they typically suffer from sluggish ion transport kinetics and poor wettability by aqueous electrolytes, resulting in unsatisfactory electrochemical performance. Here a dehydroxylation strategy is proposed to overcome the above‐mentioned limitations for polyvinyl alcohol (PVA) separators. A dehydroxylated PVA‐based membrane (DHPVA) is synthesized at a relatively low temperature in a highly concentrated alkaline solution. Part of the hydroxyl groups are removed and, as a result, the hydrogen bonding between PVA chains, which is deemed responsible for the sluggish ion transport kinetics, is minimized. At 20 °C, the ionic conductivity of DHPVA reaches 12.5 mS cm^−1^, which is almost 4 times higher than that of PVA. Additionally, DHPVA effectively promotes uniform Zn deposition, leading to a significantly extended cycle life and reduced polarization, both in a/symmetric (Cu/Zn and Zn/Zn) and full cells (Zn/NaV_3_O_8_). This study provides a new, effective, yet simple approach to improve the performance of ZMBs.

## Graphical abstract

The sluggish ion transport kinetics of PVA arises from the strong hydrogen bonding between the polymer chains. A dehydroxylated PVA (DHPVA) membrane is designed to enable high ionic conductivity, boosted charge transfer, and fast interfacial and diffusion kinetics. As a result, electrochemical performances are demonstrated in Zn/NaV₃O₈ and Zn/I_2_ full cells.

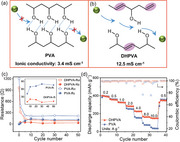

## 1 Introduction

**Figure 1:**
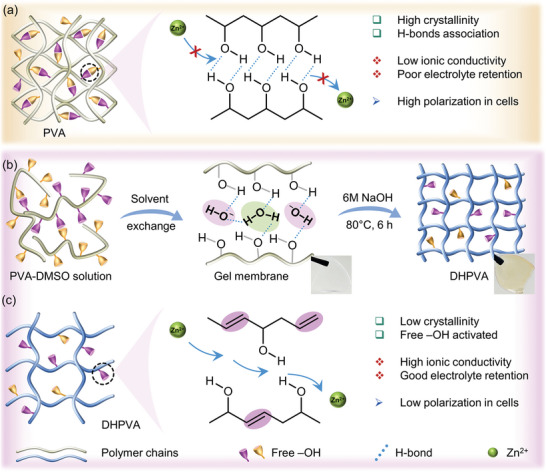
a) Zn^2+^ transport mechanism in PVA. b) Schematic diagram of DHPVA synthesis. c) Zn^2+^ transport mechanism in DHPVA.

Rechargeable batteries play a key role in grid‐scale energy storage systems to mitigate the intermittent nature of renewable energy sources such as wind and solar.^[^ ^1^ ^]^ ZMBs are particularly promising for this application, due to their inherent safety, cost‐effectiveness and environmental friendliness,^[^ ^2^ ^]^ mostly arising from the low‐cost Zn metal anode and non‐flammable aqueous electrolyte.^[^ ^3^ ^]^ However, challenges must be addressed to bridge the gap between fundamental research and industrially relevant ZMBs. These include the limited stability and capacity of cathode materials, the narrow electrochemical window of aqueous electrolytes, and the reversibility of the Zn anode.^[^ ^4^ ^]^ The separator is also a fundamental component of ZMBs, which must prevent direct contact between anode and cathode while allowing the efficient transport of ions. Currently, commercial GF membranes are widely utilized as separators in ZMBs due to their high ionic conductivity and excellent electrolyte uptake and retention. As a result, low voltage polarization is observed during battery cycling.^[^ ^5^ ^]^ However, GF separators are mechanically fragile, Zn dendrites originating on the metal anode can easily propagate through the separator and cause a battery short circuit (Figure S1a–c, Supporting Information).^[^ ^6^ ^]^ In this regard, polymer‐based separators are emerging as a promising alternative due to their superior mechanical strength which can hinder dendrite puncture and, thus, avoid cell failure caused by short circuits.^[^ ^7^ ^]^ Due to their exceptional hydrophilicity, polymers such as PVA, polyacrylamide (PAM) and polyacrylic acid (PAA) are commonly used to fabricate separators for aqueous batteries.^[^ ^8^ ^]^ Among them, PVA features excellent mechanical strength and remarkable film‐forming capability, making it a promising material for designing high‐performance separators for ZMBs. However, the application of PVA separators is impeded by the relatively sluggish ion transport kinetics, resulting from the hydrogen bonding between PVA chains.^[^ ^9^ ^]^ In fact, despite the high tensile strength of PVA (2.8 MPa, Figure S1d, Supporting Information) and the extended lifespan compared to the GF separator (Figure S1e, Supporting Information), Zn/Zn cells with a PVA separator display quite large voltage polarization (≈0.27 V) due to the high energy barrier for Zn^2+^ transport, as already demonstrated in previous literature as well.^[^ ^10^ ^]^ Indeed, the ionic conductivity of PVA is almost 4 times lower than that of GF (Figure S1f, Supporting Information). Therefore, it is of great interest to improve the ion transport kinetics of PVA to obtain a separator with great mechanical strength to suppress dendrite growth but ionic conductivity comparable with the conventional GF. The low ionic conductivity of PVA originates from the abundance of hydroxyl (‐OH) groups, which form strong hydrogen bonding between the polymer chains (**Figure** **1**a). Such bonds are responsible for the high tensile strength of PVA, however, also create a barrier for the ion transport along the polymer chains.^[^ ^11^ ^]^ To overcome this, we propose here a dehydroxylation strategy to reduce the number of ‐OH groups and, therefore, disrupt the interchain bonding network. PVA was treated in a highly concentrated alkaline solution under mildly elevated temperature (Figure 1b). During such treatment, some of the ‐OH groups are removed and new C = C bonds are formed within the polymer chains. The reduction of ‐OH groups weakens the interactions between polymer chains, promoting rapid ion transport (Figure 1c). As a result, the ionic conductivity of DHPVA reaches 12.5 mS cm^−1^ (Figure S2, Supporting Information), which is almost 4 times higher than that of PVA. Although the ionic conductivity of DHPVA is slightly lower than that of GF, it is comparable, and in most cases superior, to that of other polymer‐based separators (see Table S1, Supporting Information). In term of mechanical properties, however, the tensile strength of DHPVA is 3 times larger than that of GF (Figure S3, Supporting Information). The high ionic conductivity and good tensile strength of DHPVA anticipate its strong potential for application in high‐performance ZMBs, which will be demonstrated in the following sections.

## 2 Results and Discussion

### 2.1 Synthesis and Characteristics of a DHPVA Separator

**Figure 2:**
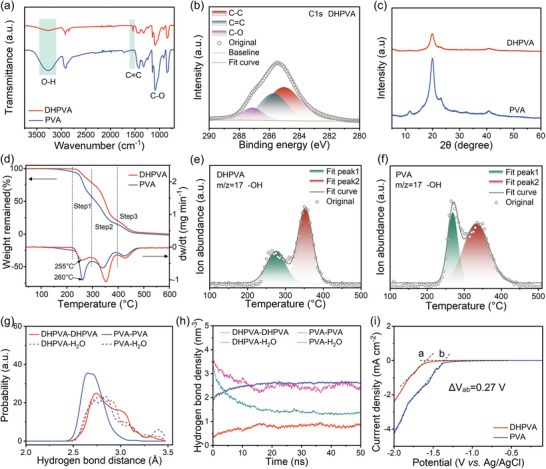
Characterization of DHPVA separator. a) FT‐IR spectra. b) C1s XPS spectrum of DHPVA. c) XRD diffractograms. d) TGA curves (left) and derivative thermogravimetric (DTG, right). ‐OH fragments from e) DHPVA and f) PVA as measured by MS in He atmosphere (the two samples have the same initial mass). g,h) HBs in DHPVA and PVA calculated by MD simulations. i) Hydrogen evolution onset in various separators measured by LSV at a scan rate of 0.2 mV s^−1^ in Ti/Ti symmetric cells with 1 M Na_2_SO_4_ electrolyte (pH=4.2).

The detailed synthesis procedure for DHPVA can be found in the experimental section of the Supporting Information. The precursor membrane was synthesized via a solvent exchange method in an alkaline solution, followed by dehydroxylation. During the dehydroxylation process, the as‐synthesized precursor membrane was heated in a highly concentrated alkaline solution of 6 M NaOH. The fabrication of DHPVA separators employs straightforward and well‐established methods, including casting, solvent exchange, and mild heat treatment, all of which are easily adaptable to industrial‐scale production. To confirm the successful dehydroxylation of PVA, characterization techniques such as Fourier‐transform infrared (FT‐IR) spectroscopy, X‐ray photoelectron spectroscopy (XPS), and X‐ray diffraction (XRD) analysis were employed. As shown in **Figure** **2**a, a new peak located at 1541 cm^−1^ ascribed to C = C bonds emerges in the FT‐IR spectrum of DHPVA, resulting from the partial removal of ‐OH groups in the polymer chains. In addition, the C‐O peak in DHPVA shows a blue shift (Figure S4, Supporting Information) compared with PVA, indicating that in DHPVA the C‐O groups are engaged in less hydrogen bonding. Consistent with the FT‐IR results, the C1s XPS spectrum of DHPVA also exhibits a peak related to C = C bonds at 285.7 eV (Figure 2b), which could not be found in PVA (Figure S5, Supporting Information). XRD analysis of dry DHPVA and PVA reveals a noticeable characteristic peak at 19.9° (Figure 2c). Although DHPVA displays a similar peak position to PVA, the intensity is significantly reduced, indicating decreased crystallinity as a result of the reduction of ‐OH groups. These results demonstrate the success of the proposed dehydroxylation strategy.

Thermogravimetric analysis (TGA) coupled with mass spectrometry (MS) shows the different thermal behavior and stability of DHPVA and PVA, as displayed in Figure 2d–f. Both DHPVA and PVA possess three distinct weight‐loss steps (Figure 2d). The initial two mass losses (step 1 and step 2) are associated with the progressive dehydration of polymeric chains and the formation of polyene structure (─C═C─C═C─).^[^ ^12^ ^]^ Interestingly, the decomposition temperature of DHPVA (255 °C) is slightly lower than that of PVA (260 °C), suggesting a weaker interaction in the polymer chains in DHPVA. From the MS results (Figure 2e,f), it is evident that the overall ‐OH signal arising from DHPVA is lower than that from PVA, as evidenced by the peak area ratio (DHPVA: PVA = 1:1.3), indicating that the ‐OH amount in DHPVA is ≈23% lower than in PVA. Molecular dynamics (MD) simulations were carried out to model the hydrogen bonds (HBs) network in the two samples (Figure S6, Supporting Information). The distance and density of HBs between DHPVA‐DHPVA, PVA‐PVA, DHPVA‐H_2_O, and PVA‐H_2_O are shown in Figure 2g,h. DHPVA displays a broader range of HBs distances between molecular chains, and the major peak shifts to a higher distance of 2.7 Å than that of PVA (2.6 Å), indicating DHPVA has less interchain HBs compared to PVA. However, DHPVA is more inclined to form HBs with H_2_O (DHPVA‐H_2_O), as shown in Figure 2h, in which DHPVA‐H_2_O shows higher HBs density than PVA‐H_2_O. Therefore, the activity of H_2_O in DHPVA is likely reduced, anticipating a high potential to suppress hydrogen evolution reaction (HER).^[^ ^13^ ^]^ To confirm this, the effect of the DHPVA on the HER was characterized using linear sweep voltammetry (LSV) in a three‐electrode cell configuration with a 1 M Na_2_SO_4_ solution at the same pH (4.2) of the 1 m Zn(CF_3_SO_3_)_2_ electrolyte. The absence of Zn^2+^ in the electrolyte can exclude the influence of Zn deposition, whose redox potential is close to that of HER, thus allowing a reliable in‐depth investigation of the HER during the negative scan. As shown in Figure 2i, DHPVA exhibits a significantly lower HER onset potential (ΔV = 0.27 V) than PVA, which is consistent with MD simulations. Overall, it is demonstrated that the dehydroxylation significantly alters the hydrogen bonding characteristics of DHPVA. The less interchain hydrogen bonding facilitates ion transport, while the more hydrogen bonding with H_2_O contributes to the suppression of hydrogen evolution.

### 2.2 Investigation on the Kinetics of Zn Plating with a DHPVA Separator

**Figure 3:**
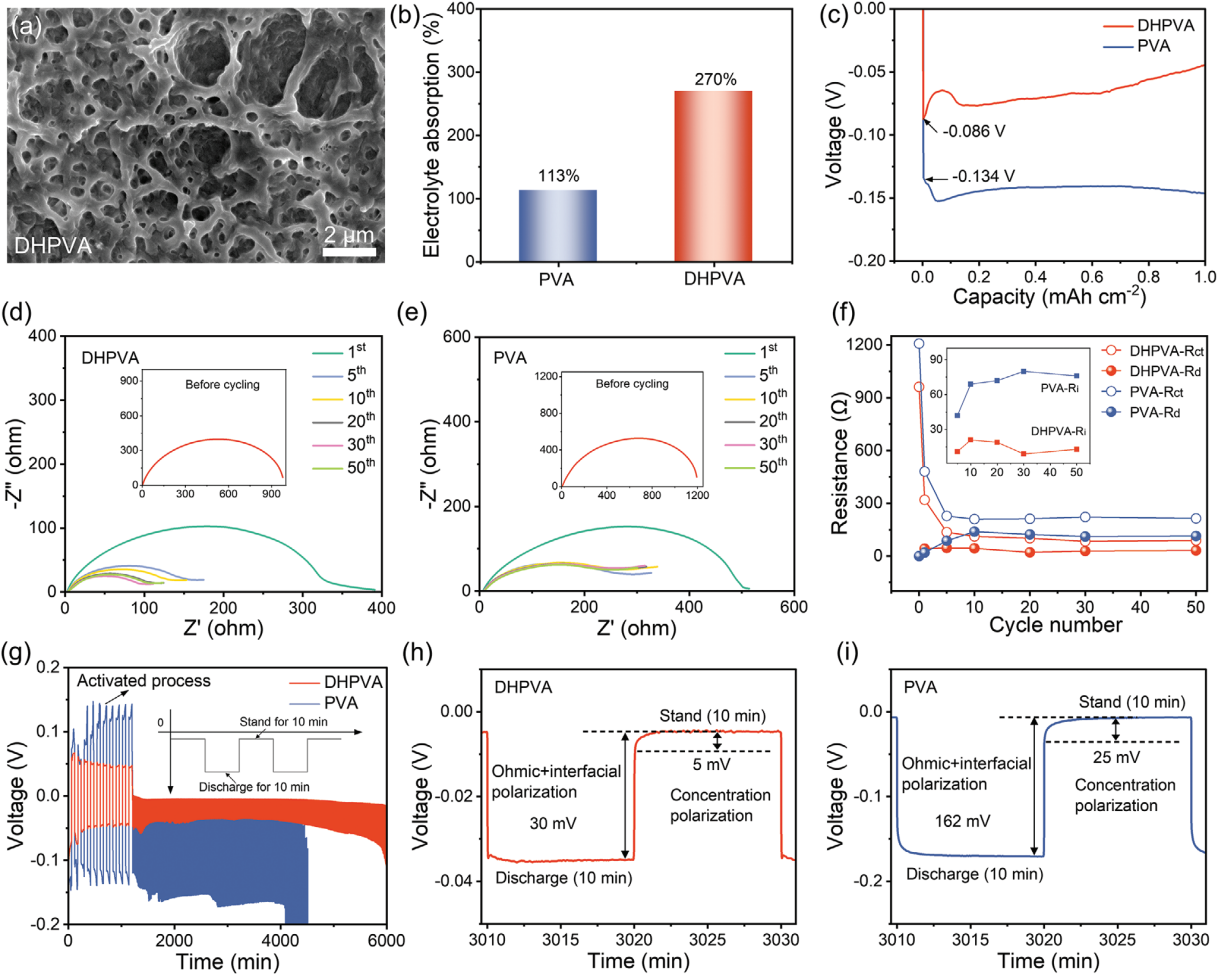
Study of Zn deposition with a DHPVA separator. a) Top‐view SEM image of a DHPVA separator. b) Electrolyte absorption of different separators. c) Voltage‐deposition capacity profiles of Zn/Cu cells under a constant current density of 1 mA cm^−2^. d,e) EIS evolution of Zn/Zn cells after different cycles at 1 mA cm^−2^/1 mAh cm^−2^ (the inserts are the EIS plots before cycling). f) Resistance values at different cycles extrapolated by DRT analysis. g) Intermittent discharge at 1 mA cm^−2^ after a preconditioning 10 cycles and corresponding enlarged curves for cells with h) DHPVA and i) PVA.

To understand the influence of different separators on the Zn plating process and its kinetics, the morphological characteristics of DHPVA and PVA were first investigated, since these features play an important role in ion transport, especially the structure and distribution of pores in the membrane.^[^ ^14^ ^]^ As shown in **Figure** **3**a and Figure S7a (Supporting Information), DHPVA features a porous structure with interconnected macropores, which is beneficial for electrolyte adsorption and retention (Figure 3b). This kind of porous structure also provides abundant channels for ion transfer. In contrast, PVA shows a dense and flat surface with no evident porosity (Figure S7b, Supporting Information), leading to poor electrolyte uptake and sluggish ion transport. The difference in pore structure significantly influences ion transport dynamics and, consequently, overall battery performance. To confirm this, both Zn/Cu and Zn/Zn cells were assembled and tested. Zn/Cu cells with DHPVA separator display a substantially lower initial nucleation overpotential (0.086 V) compared to that of PVA cells (0.134 V), as shown in Figure 3c. Moreover, while it remains constant with PVA, the growth overpotential keeps decreasing with increased deposition capacity in DHPVA cells. These results indicate that the DHPVA can effectively improve the nucleation and growth of Zn.

In addition, the electrochemical impedance of Zn/Zn cells with DHPVA and PVA separators was further evaluated. The electrochemical impedance spectroscopy (EIS) data after different cycles, as shown in Figure 3d,e, were further analyzed using the distribution of relaxation times (DRT) method, with the corresponding results shown in Figure S8 (Supporting Information). The DRT method is highly effective in distinguishing various polarization losses, including diffusion resistance (R_d_), interfacial resistance (R_i_) and charge‐transfer resistance (R_ct_). These resistances are analyzed across different frequency ranges, allowing for a detailed breakdown of the kinetic processes in the battery system.^[^ ^15^ ^]^ Peaks in the DRT plot reflect polarization resistances associated with distinct relaxation times (τ). Based on previous studies, τ_1_, τ_2_, and τ_3_ represent the resistance of the diffusion process (R_d_), the resistance of the electrode/electrolyte interphase (R_i_) and that of the charge‐transfer process (R_ct_), respectively.^[^ ^16^ ^]^ Figure 3f compares the polarization resistances of cells using DHPVA and PVA separators. For DHPVA cells, R_ct_ dramatically decreases from 962 to 89 Ω after 50 cycles, while R_d_ remains relatively constant at 32 Ω. For PVA cells, R_ct_ drops from 1208 to 214 Ω after 50 cycles, but R_d_ increases significantly to 114 Ω. Moreover, a higher interface resistance (R_i_) in PVA cells (76 Ω) compared to DHPVA cells (13 Ω) after 50 cycles evidences a deteriorating electrolyte‐electrode interface. These results suggest that the DHPVA separator can comprehensively improve the kinetics of all processes: charge transfer, interfacial transport, and diffusion; providing a solid foundation for achieving high‐performance ZMBs.

The voltage polarization is also an important indicator for the Zn^2+^ interfacial kinetics, which includes ion‐charge transfer and deposition kinetics. The voltage polarizations of Zn/Zn cells were investigated by an intermittent discharge process with constant current.^[^ ^17^ ^]^ As shown in Figure 3g–i, after activating the cells for 10 full plating/stripping cycles at a current density of 1 mA cm^−2^ and areal capacity of 1 mAh cm^−2^, they were repeatedly discharged for 10 min at the same current density, followed by a 10 min rest period in the following cycles. Polarization occurs mainly due to ohmic resistance and interfacial polarization, resulting in a voltage drop during the discharge process. Additionally, concentration polarization is also observed during the rest period due to the redistribution of ions within the electrolyte and the electrodes. Both ohmic and concentration polarizations are significantly reduced in DHPVA cells, with ≈30 and 5 mV for ohmic and concentration polarization, respectively, which are much lower than that in PVA cells (≈162 and ≈25 mV for ohmic and concentration polarization, respectively). Throughout the whole discharge process, the DHPVA cells can continuously discharge for 40 h, showing a depth of discharge (DOD) of 81%, whereas the PVA cells only discharge for 23 h with a DOD of 47%. These results reveal that the DHPVA separator enhances the reaction kinetics of the Zn electrode, highlighting its potential for improving the performance of ZMBs.

### 2.3 Electrochemical Performances Verification

**Figure 4:**
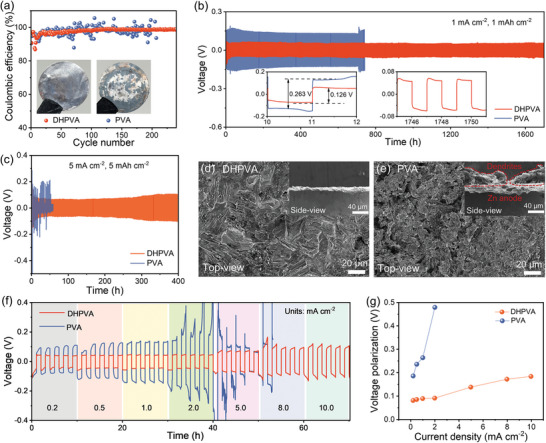
Electrochemical behavior of Zn/Cu and Zn/Zn cells with DHPVA and PVA separators. a) CE of Zn/Cu cells at 1 mA cm^−2^ (the inserts are the photos of Cu electrodes with Zn deposits after 10 cycles). Cycling performance of Zn/Zn cells b) at 1 mA cm^−2^/1 mAh cm^−2^ and c) 5 mA cm^−2^/5 mAh cm^−2^. Morphology of Zn anodes after cycling with d) DHPVA and e) PVA at 1 mA cm^−2^/1 mAh cm^−2^. f) Rate performance of Zn/Zn cells under different current densities and g) corresponding polarization.

The enhanced reaction kinetics enabled by the DHPVA separator are expected to lead to improved reversibility of the Zn electrode, which can be verified in Zn/Cu cells. As shown in **Figure** **4**a and Figure S9 (Supporting Information), DHPVA cells exhibit a smaller voltage gap (0.076 V), and the Coulombic efficiency (CE) proceeding an initial fluctuation starts increasing slightly up to 99.3% after 135 cycles, and then it stabilizes, indicating reversible and stable Zn plating/stripping. In contrast, PVA cells display a fluctuating CE, and a larger voltage gap (0.22 V) is also observed, suggesting higher resistance and less efficient plating/stripping. The homogeneity of the Zn deposited on Cu foil in different cells was verified first by visually inspecting the surface of the Cu foils after cycling. With DHPVA, the silver Zn is uniformly distributed on the surface of the Cu foil, whereas a heterogeneous surface morphology is clearly evident when using PVA. The corresponding SEM images reported in Figure S10 (Supporting Information) confirm the same.

The Zn/Zn cells with DHPVA also demonstrate superior performance with a long lifespan of 1750 h at a current density of 1 mA cm^−2^/1 mAh cm^−2^, outperforming its PVA counterpart (750 h) (Figure 4b). In addition, the voltage polarization of DHPVA cells is much smaller than that of PVA cells. Even at a high current density of 5 mA cm^−2^/5 mAh cm^−2^, DHPVA cells still achieve a cycle lifespan of over 400 h (Figure 4c), while, the PVA cells fail after just 1 cycle (Figure S11, Supporting Information). The morphology evolution of Zn deposition was further investigated by using SEM. The surface of the Zn anode with DHPVA shows a specific texture with platelets compactly aligned, and the cross sectional image reveals a flat and dense edge after cycling for 270 h at 1 mA cm^−2^/1 mAh cm^−2^ (Figure 4d). In contrast, irregular particles composed of Zn dendrites and by‐products from side reactions are observed on the Zn anodes cycled with PVA (Figure 4e). Furthermore, the rate performance of the symmetrical cells was tested by stepwise increasing current densities from 0.2 to 10 mA cm^−2^ (Figure 4f). At each current density, five cycles are conducted with a fixed deposition or dissolution time of 1 h. DHPVA cells are able to operate at a challenging testing condition of 10 mA cm^−2^/10 mAh cm^−2^, while PVA cells fail when the current density increases to 2 mA cm^−2^ due to the sluggish ion transport kinetics and severe Zn dendrite formation. As previously shown, owing to the fast kinetics, DHPVA cells show a low voltage polarization at all current densities. On the contrary, PVA cells show a significant increase in voltage polarization with increasing current density (Figure 4g). This result suggests that the superior features of DHPVA, such as its enhanced ionic conductivity and optimized hydrogen bonding network, reduce the energy barriers for Zn nucleation at all investigated current densities. The lower nucleation and growth overpotential when increasing the current density highlights the stable and efficient ion transport characteristics of DHPVA, further supporting its superior performance as a separator in ZMBs.

**Figure 5:**
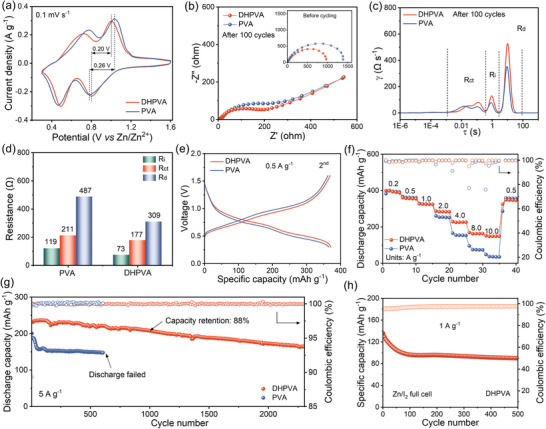
Electrochemical performance of Zn/NaV_3_O_8_ full cells. a) CV plots at a scan rate 0.1 mV s^−1^. b) EIS spectra before cycling and after 100 cycles. c) DRT profiles and d) corresponding resistances in each process. e) Galvanostatic charge and discharge curves of the second cycle at 0.5 A g^−1^. f) Rate performance. g) Cycling performance at 5 A g^−1^ (5 cycles for activating at 0.5 A g^−1^). h) Cycling performance of the Zn/I_2_ full cell at 1 A g^−1^.

The performance of the DHPVA separator was also assessed in full‐cell configuration with typical NaV_3_O_8_•1.5H_2_O as cathode material coupled with Zn metal anode. The synthesis method and characteristics of the cathode materials are shown in the experimental section of Supporting Information and Figure S12 (Supporting Information). Cyclic voltammetry (CV) measurements were performed to investigate the electrochemical behavior of the full cells (**Figure** **5**a). Two pairs of reduction/oxidation peaks are observed for both the DHPVA‐ and PVA‐containing cells, corresponding to the valence transitions from V^5+^ to V^4+^ and from V^4+^ to V^3+^, respectively, which are associated with Zn^2+^ insertion and extraction into/from the cathode.^[^ ^18^ ^]^ DHPVA cells show a smaller voltage hysteresis (0.2 V) compared to PVA cells (0.26 V), indicating more efficient electrochemical processes. When the scan rates increase from 0.1 to 2.0 mV s^−1^, the CV curves of DHPVA cells always show sharp and well‐defined peaks, suggesting the superior reversibility of the redox reactions (Figure S13a, Supporting Information). However, the peaks in the voltammogram of PVA cells become broader and shift as the scan rate is higher than 0.8 mV s^−1^, due to high resistance and sluggish ion transport kinetics (Figure S13b, Supporting Information). The EIS spectra collected before and after cycling, as shown in Figure 5b, were further analyzed using the DRT method, with the corresponding results shown in Figure S14 (Supporting Information) and Figure 5c,d. Before cycling, the R_ct_ of DHPVA and PVA cells are 1374 and 961 Ω. Interestingly, an additional process with very small time τ is also observed in the fresh cells, which may be associated with contact issues (Figure S14, Supporting Information). The related contact resistance (R_c_) shows initial values of 21 Ω for DHPVA and 13 Ω for PVA. After 100 cycles, such a feature reduces due to the improved interface contact during repeated charge‐discharge processes (Figure 5c). On the other hand, additional features related to R_ct_, R_i_ and R_d_ evolve upon cycling, which are always significantly low in DHPVA cells (Figure 5d). These results are consistent with the kinetics analysis of the Zn/Zn cells, confirming that DHPVA effectively minimizes polarization resistances in full cells as well.

The cycling performance was evaluated under galvanostatic cycling at different current densities as well. As shown in Figure 5e, DHPVA cells achieve a high reversible capacity of 371 mAh g^−1^ at a current density of 0.5 A g^−1^, which is one of the best results obtained compared to the literature.^[^ ^19^ ^]^ The rate capability is shown in Figure 5f, the discharge capacities of DHPVA cells are 398, 357, 325, 284, 225, 163, and 148 mAh g^−1^ at the current densities of 0.2, 0.5, 1, 2, 4, 8, and 10 A g^−1^, respectively. The reversible capacity difference between DHPVA and PVA cells is negligible at relatively low current densities (< 1.0 A g^−1^), but it becomes very pronounced at high current densities over 2 A g^−1^ due to the fast capacity decay of PVA cells with the increase of current density. In fact, the reversible capacity of PVA cells at 10 A g^−1^ is only 37 mAh g^−1^. This result relates to the sluggish transport kinetics through the PVA separator, leading to a higher ohmic drop of the PVA cells. As mentioned before, since the ionic conductivity of the PVA separator is limited, the rate of Zn^2+^ diffusion is not sufficient at increased current densities. As a result, fast capacity decay is observed. In addition, the morphology of Zn anodes cycled at a current density of 2 A g^−1^ was analyzed (Figure S15, Supporting Information). The Zn anode cycled in the cell with the DHPVA separator displays a dense and flat surface, while a layer of loose and coarse island‐like deposits is observed on the Zn anode of the cell with PVA, probably resulting in “dead Zn”.^[^ ^20^ ^]^ In terms of the long cycling performance, as shown in Figure 5g, the capacity of PVA cells rapidly declines to 147 mAh g^−1^ after 600 cycles at a current density of 5 A g^−1^, ending up with cell failure (Figure S16, Supporting Information). In contrast, DHPVA cells retain a high initial capacity of 224 mAh g^−1^ with a capacity retention of 88% after 1000 cycles, which also exhibits much lower voltage polarization (Figure S17, Supporting Information). The performance of DHPVA in the Zn/NaV_3_O_8_ full cell is comparable to other polymer‐based separators in full cells with vanadium‐based cathodes (Table S2, Supporting Information). Additionally, the performance of DHPVA separator in another full‐cell configuration, such as the Zn/I_2_ battery system, was also evaluated. As shown in Figure S18a (Supporting Information), the CV profiles of DHPVA full cells collected at 1 mV s^−1^ in the voltage range from 0.6 to 1.8 V exhibit a pair of reversible redox peaks ≈1.4/1.0 V (vs Zn/Zn^2+^), corresponding to the redox reaction between I_2_/I^−^. Furthermore, the DHPVA cell also shows good cycling stability (Figure 5h) and rate performance (Figure S18b, Supporting Information), highlighting the broad applicability of the DHPVA separator. These results demonstrate the effectiveness and practicality of the DHPVA separator, demonstrating its potential to advance the application and development of high‐performance ZMBs.

## 3 Conclusion

In summary, a dehydroxylation strategy has been proposed to improve the transport properties of PVA‐based separators. Part of the ‐OH groups were successfully removed from the original PVA chains to minimize the formation of inter‐chain hydrogen bonds, which is responsible for the sluggish ion transport kinetics and poor electrolyte uptake. As a result, high ionic conductivity and suppressed dendrite formation are achieved in ZMBs with the as‐designed DHPVA separator. Moreover, the resistance of each process in Zn/Zn cells, as analyzed by the DRT method, indicates that DHPVA substantially boosts charge transfer, interfacial and diffusion kinetics. Therefore, DHPVA‐containing Zn/Zn cells exhibit remarkable cycling stability with a long cycling life of 1750 h and small polarization (0.126 V). When the current density is up to 10 mA cm^−2^, DHPVA cells can be sustained without observing large polarization. In addition, Zn/NaV_3_O_8_ full cells with DHPVA demonstrate superior rate capability, with capacity recovering to 97% of the initial value (358 mAh g^−1^) upon returning the current density to 0.5 A g^−1^. Additionally, at a high current density of 5 A g^−1^, the DHPVA full cells retain a high capacity of 208 mAh g^−1^ after 1000 cycles, corresponding to 88% capacity retention. This study provides a new, effective, yet simple approach to improve the performance of ZMBs separators, thereby contributing to the further development of efficient, durable, sustainable and low‐cost energy storage systems.

## Conflict of Interest

The authors declare no conflict of interest.

## Supporting information



Supporting Information

## Data Availability

All data generated or analyzed during this study are included in this article and its Supporting Information. The data that support the findings of this study are available from the corresponding author upon request.
