# Supplementary material for: Dehydroxylated Polyvinyl Alcohol Separator Enables Fast Kinetics in Zinc‐Metal Batteries
Source: Small. 2025 Jan 26;21(9):2410758. doi: 10.1002/smll.202410758 (PMC11878257; doi:10.1002/smll.202410758)
Supplement: Supplementary file 1 — Supporting Information [file SMLL-21-2410758-s001.docx]

Supporting Information for

Dehydroxylated polyvinyl alcohol separator enables fast kinetics in zinc-metal batteries

Yao Qin, Fuhua, Yang*, Jodie A. Yuwono, Alberto Varzi*

Y. Qin, F. Yang, A. Varzi

Helmholtz Institute Ulm (HIU), Helmholtzstrasse 11, 89081, Ulm, Germany

Y. Qin, F. Yang, A. Varzi

Karlsruhe Institute of Technology (KIT), P.O. Box 3640, 76021, Karlsruhe, Germany

E-mail: fuhua.yang@kit.edu; alberto.varzi@kit.edu

J. A. Yuwono

School of Chemical Engineering, The University of Adelaide, Adelaide, SA 5005, Australia

**Experimental Section**

**Synthesis method of a DHPVA membrane**

1. Synthesis of precursor membrane by solvent exchange method: 1.5 g of polyvinyl alcohol (PVA) powder (fully hydrolyzed, Mw approx. 145,000, Sigma-Aldrich) was dissolved in 10 mL dimethyl sulfoxide (DMSO, 99.9%, VMR Chemicals) to form a viscous mixture. This mixture was cast onto a PET substrate and subsequently immersed in a 6 M NaOH (Alfa Aesar) solution. After 12 h, a precursor membrane was obtained. The optimized NaOH concentration of 6 M was chosen for the following reasons: 1) concentrations below 6 M resulted in very fragile membranes; and 2) a 6 M NaOH solution can effectively facilitate the dehydroxylation reaction (next step).

2. Dehydroxylation process: The precursor membrane was placed in a fresh 6 M NaOH solution and heated to 80°C for 6 h. A yellow DHPVA membrane was produced after repeated washing with Milli-Q water until the wash water achieved a neutral pH. The thickness of the wet DHPVA membrane is 284 μm.

**Preparation of a PVA membrane**

1.0 g of PVA powder was dissolved in 10 mL DMSO to form a viscous mixture. This mixture was then poured into PTFE dishes and heated to 80°C for 48 h. Finally, a PVA membrane was obtained. The thickness of the wet PVA membrane is 289 μm.

**Preparation of NaV_3_O_8_•1.5H_2_O Cathode^[^**^1]^

3.0 g of commercial V_2_O_5_ powder (Alfa Aesar) was added into a NaCl (VMR Chemicals) aqueous solution (2 mol L^−1^). After stirring at 30°C for 72 h, the suspension was centrifuged and washed with Milli-Q water and ethanol several times. The NaV_3_O_8_•1.5H_2_O nanowires were then obtained.

**Preparation of I_2_ cathode**

The activated carbon (YP-50F), Ketjen Black (KB), and polyvinylidene difluoride (PVDF) were combined in a mass ratio of 8:1:1. The mixture was dispersed in N-methylpyrrolidone (NMP) to form a slurry, which was subsequently coated onto carbon fiber to create the electrode layers. A solution of 1 M KI and 0.1 M I₂ (VMR Chemicals) was applied to the electrode layer, resulting in an active material loading of approximately 3 mg cm^-2^ (I^-^) for the cathode.

**Electrochemical Measurements**

The positive electrodes were composed of NaV_3_O_8_•1.5H_2_O, conducting carbon (Super C45), and PVDF at a mass ratio of 7:2:1. The slurry was dispersed in NMP, and then coated onto a Ti foil to obtain the electrode layers. The active material loading of the cathode was ca. 1.2-1.3 mg cm^-2^. Zn foil (>99.95%) with a thickness of 75 μm was supplied by Goodfellow, which was punched into disks with a diameter of 15 mm in full cells and 12 mm in a/symmetric (Zn/Cu and Zn/Zn) cells. The glass fiber (GF, Whatman GF/A), DHPVA, and PVA membranes were punched into disks (Φ = 16 mm) as separators. 1 M Zn(CF_3_SO_3_)_2_ (Alfa Aesar) aqueous solution was used as the electrolyte. The DHPVA and PVA membranes were soaked in the electrolyte before assembling the cells. All cyclic voltammetry (CV), electrochemical impedance spectroscopy (EIS), and linear sweep voltammetry (LSV) were performed using an electrochemical workstation (VMP-3 Biologic Science). Especially, the CV tests of full cells were tested in the 0.3 V to 1.6 V voltage range; the frequency range of the EIS test was 10^-2^-10^6^ Hz; the LSV was measured in three-electrodes Swagelok-type cells at a scan rate of 0.2 mV s^−1^ with a 1 M Na_2_SO_4_ solution as electrolyte, Ti foil as working and counter electrode, and leakless Ag/AgCl electrode as reference. All cells were assembled in 2032-coin cell configuration for long-term stability tests on a battery tester (MACCOR, 4000). All the electrochemical tests were conducted at 20°C.

Ionic conductivities of the separators were measured using stainless steel as blocking electrodes and calculated through the following equation^[2]^:

σ =$\frac{d}{RS}$

Where *d* represents the thickness of the separator, *R* represents the resistance according to EIS measurement, and *S* is the contact area between the separator and electrodes.

**Distribution relaxation times (DRT) method**

The DRT method can analyze the diffusion resistance (R_d_), interfacial resistance (R_i_), charge-transfer resistance (R_ct_), and contact resistance (R_c_), according to the following formula:

$$Z(f)=R_{O}+R_{P}\int_{0}^{\infty} \frac{\gamma(\tau)}{1+j2\pi f\tau}d\tau$$

Where $Z(f)$ represents EIS for fitting the DRT model, R_o_ and R_p_ represent ohmic and polarization resistance, respectively. $\gamma\left( \tau\right)$ represents the distribution function of relaxation times $(\tau)$, $j$ is the imaginary unit, $f$ is the frequency. The task of the DRT method is to identify $\gamma(\tau)$ from $Z(f)$.

This DRT implementation is based on ideas and methods introduced by Professor Francesco Ciucci's research group.^[3]^

The DRT results presented in this work were obtained using a DRT toolbox of the RelaxIS software, in which the R_d_, R_i_, R_ct_ and R_c_ were calculated by integrating the peak areas corresponding to different $\tau$ ranges.

**Characterizations Method**

Morphologies of the separators, cathode materials, and Zn anodes were characterized by a scanning electron microscope (SEM, ZEISS Crossbeam XB340). Information on the chemical composition of the materials was collected by VERTEX 70v vacuum Fourier transform infrared (FT-IR) spectrometer. X-ray diffractograms (XRD) were recorded on a Bruker D8 Advance diffractometer with Cu Kα radiation. Thermogravimetric analysis was achieved by TGA-Netzsch equipped with mass spectrometry (MS). X-ray photoelectron spectroscopy (XPS) was carried out on a Phoibos 150 XPS spectrometer (SPECS) with a Dealy Line Detector (Surface Concept). The stress-strain curves of various separators were obtained by using an Instron 5565A tester, all separators were in a wet state when tested.

**Computational Methodology**

All molecular dynamics (MD) simulations were performed using the GAFF2 force field.^[4]^ The ACPYPE was employed to obtain the GAFF2 force field topology.^[5]^ The simulation box size of 6×6×6 nm^3^ was used in all simulation models. The simulation systems consist of Zn^2+^, CF_3_SO_3_^-^, and H_2_O, with/without PVA or DHPVA molecules. The models of PVA and DHPVA molecules with 13 carbon atoms long were used. The ratio of each component is shown in Table S1. The cut-off distance of 1.2 nm was used for Lennard-Jones potential. The Coulombic potential was measured using Particle Mesh Ewald (PME) with a cut-off distance of 1.2 nm and Fourier grid spacing of 0.12. All bonds were constrained with LINCS algorithm. Periodic boundary conditions were applied in all directions. The MD simulations were started by running initial energy minimization, followed by 1000 ps of NVT simulation and 1000 ps of NPT simulation with an integration time step of 0.001 ps. All the simulation systems were finally maintained at 298 K using the Nose-Hoover thermostat for 50 ns to collect simulation data. A time constant of 1 ps was applied for the temperature coupling.


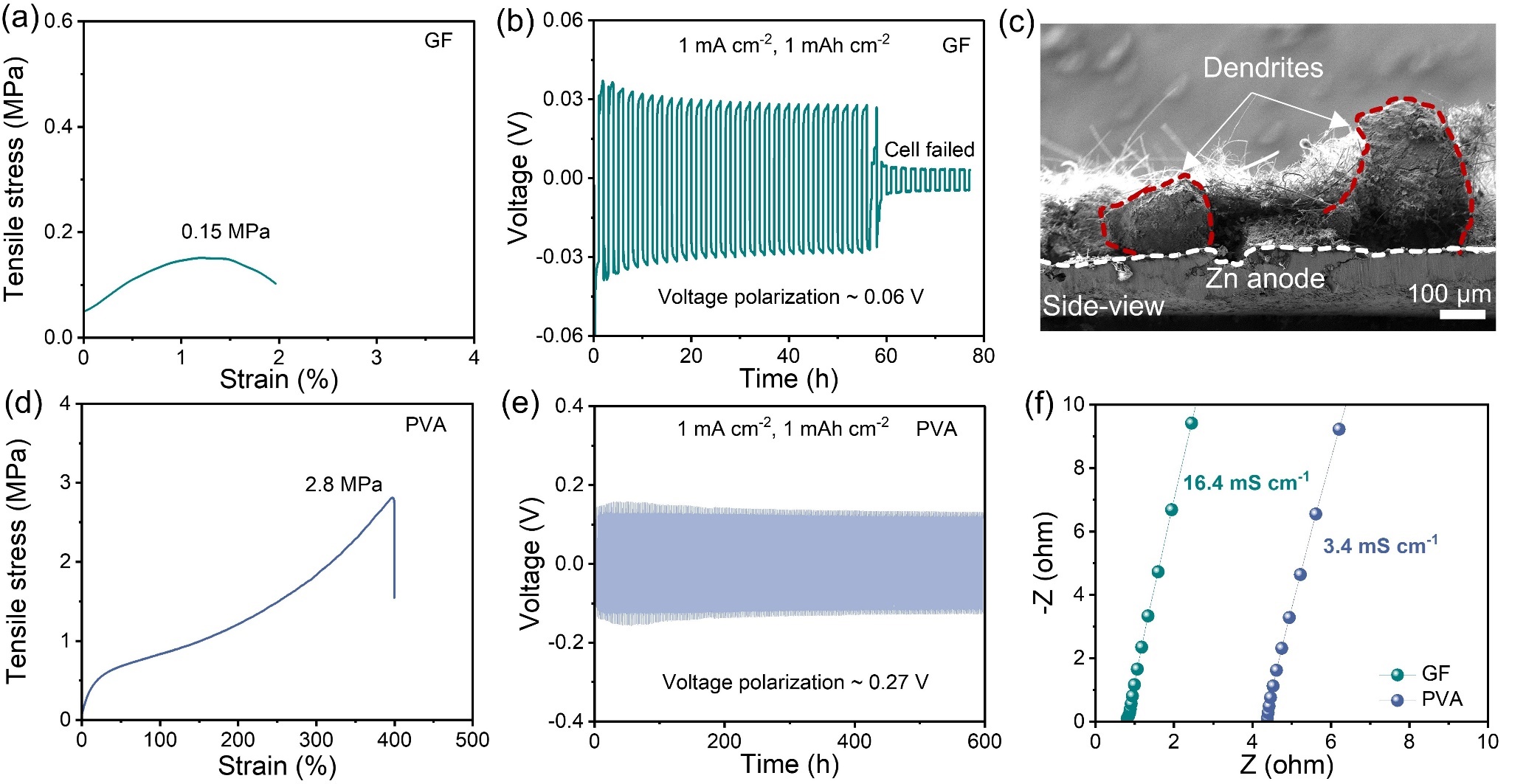


**Figure S1.** Advantages and disadvantages of commercial GF and PVA separators. (a) Stress-strain curve of GF. (b) Cycling performance of a Zn/Zn cell with GF at 1 mA cm^-2^/1 mAh cm^-2^ and (c) the corresponding cross-section scanning electron microscope (SEM) image of the Zn anode. (d) Stress-strain curve of PVA. (e) Cycling performance of a Zn/Zn cell with PVA at 1 mA cm^-2^/1 mAh cm^-2^. (f) Nyquist plots of GF and PVA with two stainless blocking electrodes and the calculated ionic conductivity. All measurements are performed at 20°C.

**Figure S2.** Nyquist plots of DHPVA with two stainless blocking electrodes and the calculated ionic conductivity.

**Figure S3.** Stress-strain curve of DHPVA.

**Figure S4.** Enlarged FT-IR curves in the range of 1200-1000 cm^-1^.

**Figure S5.** C1s XPS spectrum of PVA.

Zn^2+^

CF_3_SO_3_^-^

H_2_O


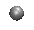

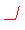

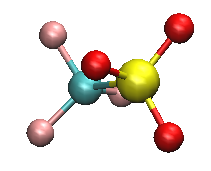

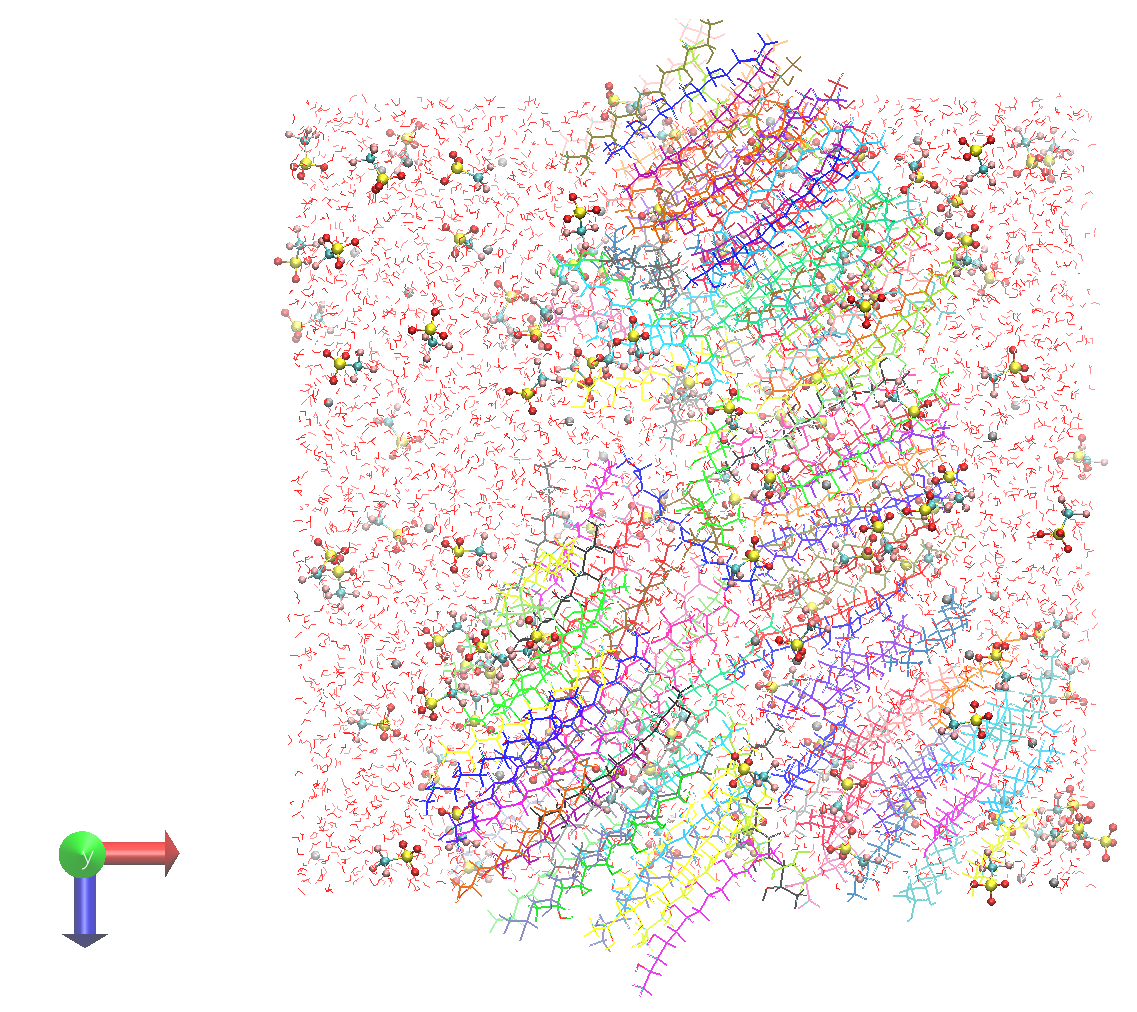

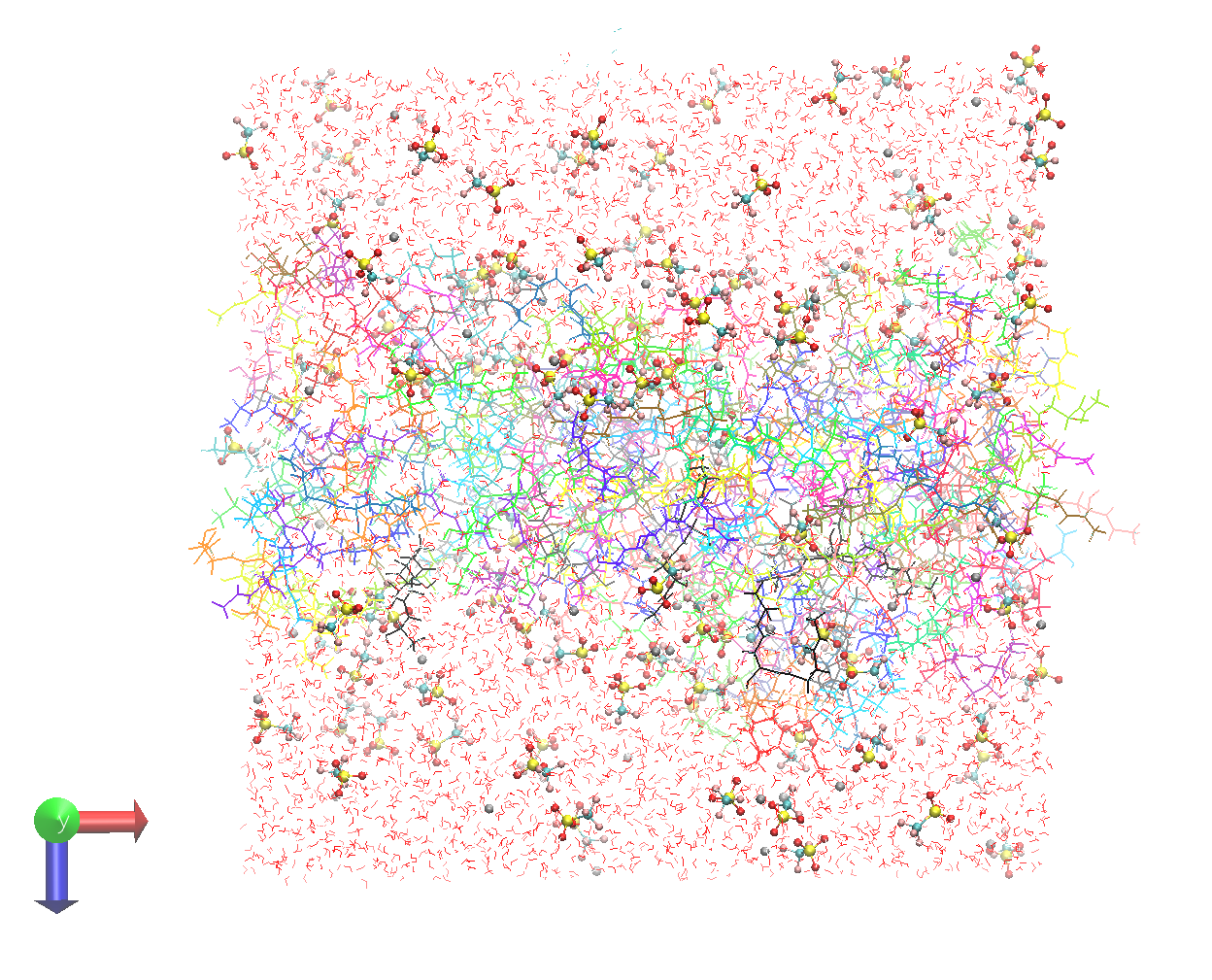


(b)

DHPVA

PVA

(a)

| Electrolyte components | Number of molecules | |
| --- | --- | --- |
|  | PVA | DHPVA |
| Zn^2+^ | 50 | 50 |
| CF_3_SO_3_^-^ | 100 | 100 |
| PVA/DHPVA | 128 | 182 |
| H_2_O | 5560 | 5560 |

(c)

**Figure S6.** The models of MD simulations, (a) PVA, (b) DHPVA, and (c) ratio of each component.


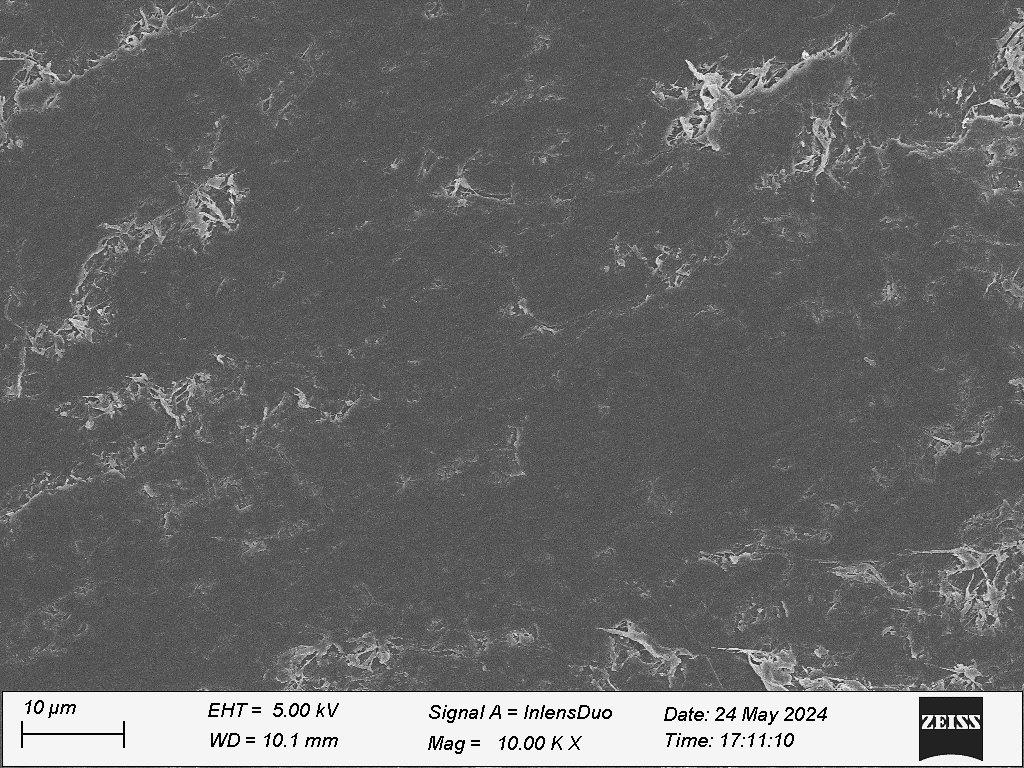


10 μm


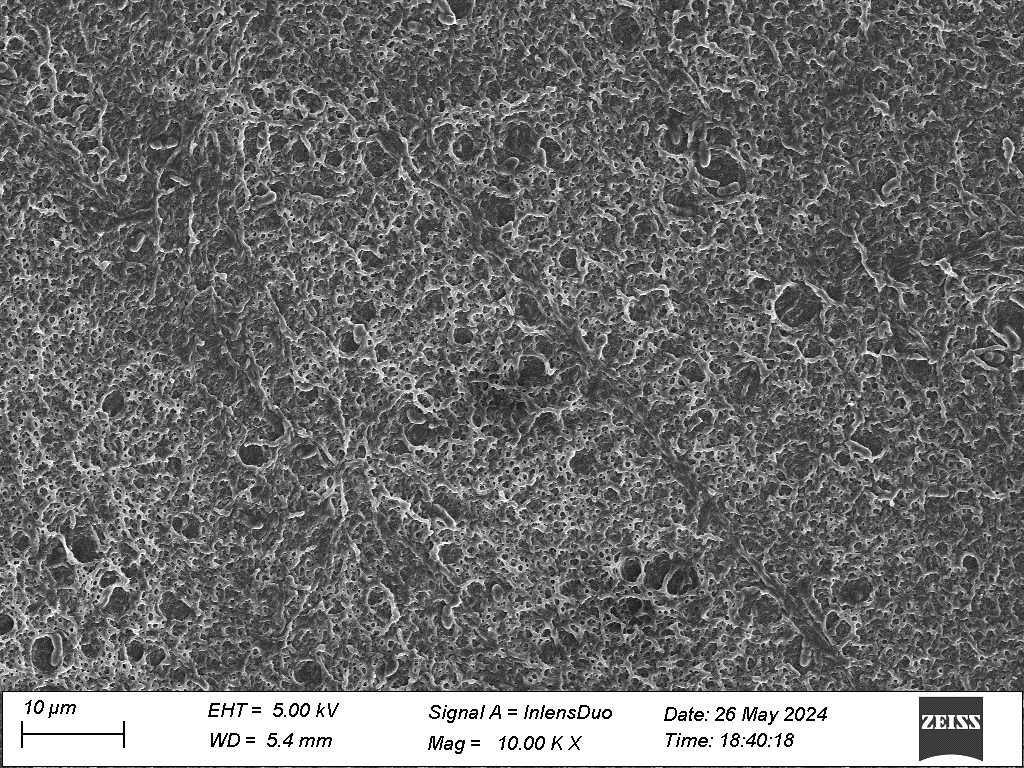


10 μm

(a)

(b)

DHPVA

PVA


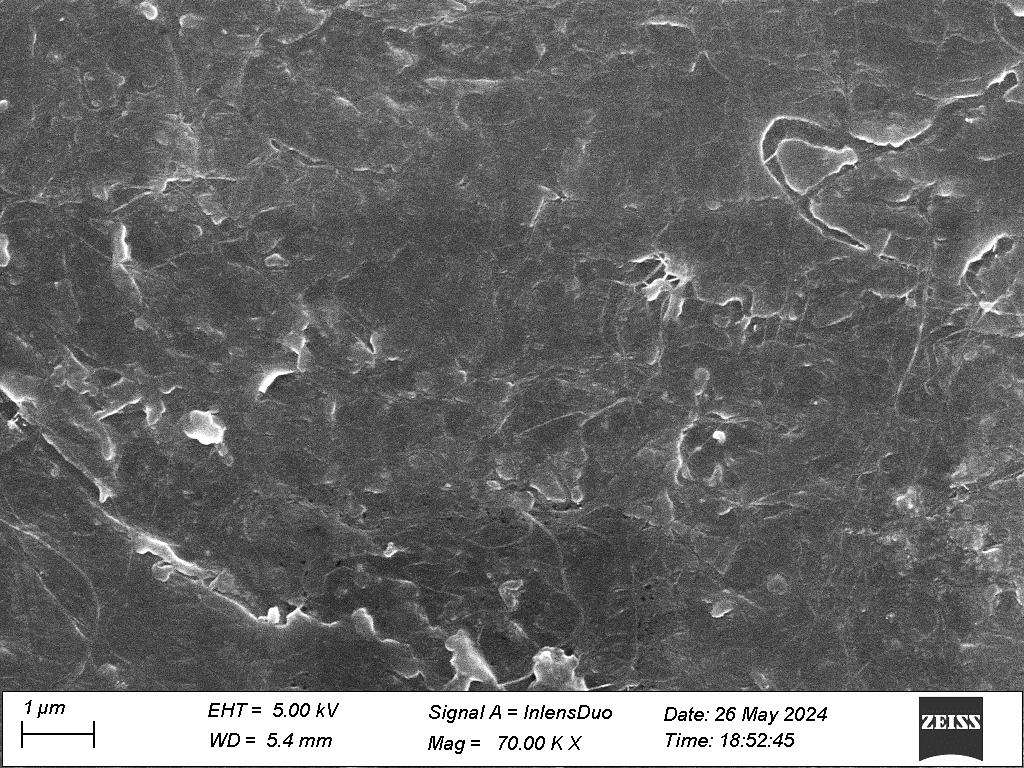


2 μm

**Figure S7.** Top-view SEM image of (a) DHPVA and (b) PVA.

**Figure S8.** DRT profiles at different cycles. (a) the 1^st^, (b) 5^th^, (c) 10^th^, (d) 20^th^. (e) 30^th^, (d) 50^th^.


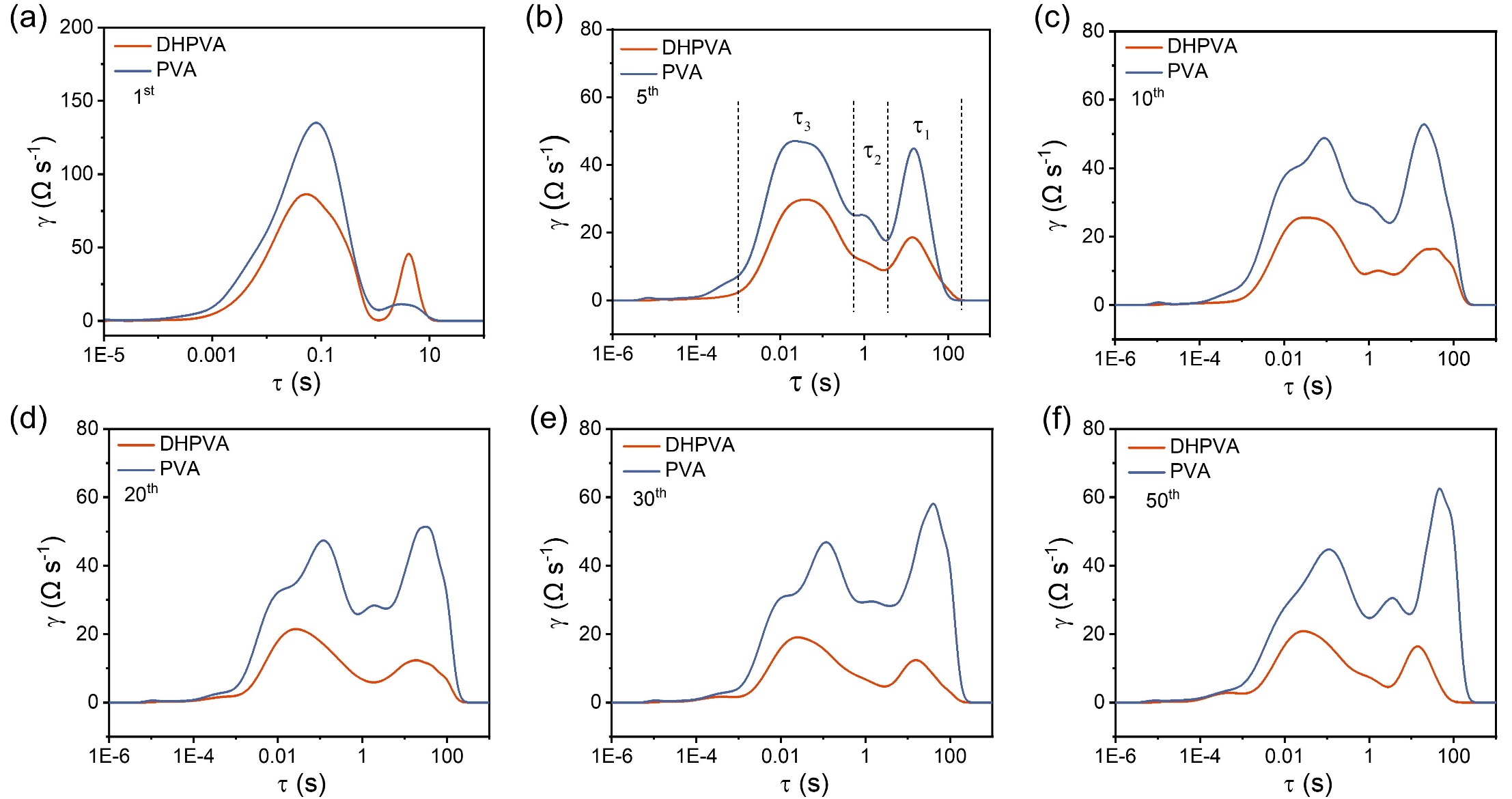


**Figure S9.** Voltage-capacity profiles of Zn/Cu cells with (a) DHPVA and (b) PVA at 1 mA cm^-2^/1 mAh cm^-2^.

(a)

(b)

**Figure S10.** SEM images of Cu foil with Zn deposits after 10 cycles at 1 mA cm^-2^, (a) with PVA separator and (b) with DHPVA separator.


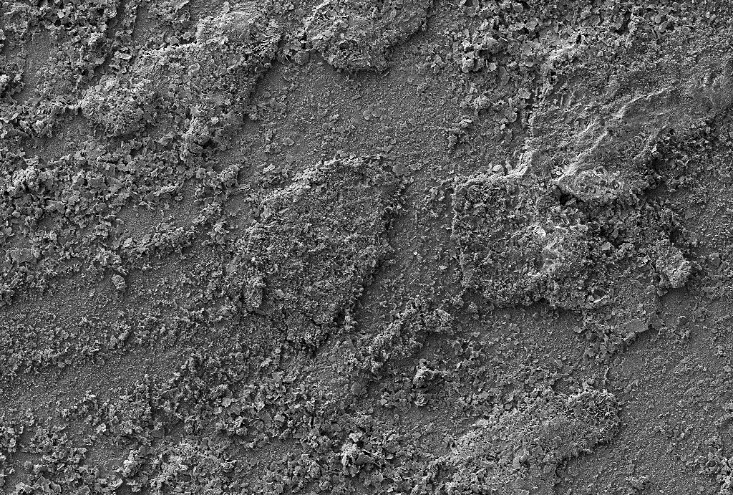


80 μm

80 μm


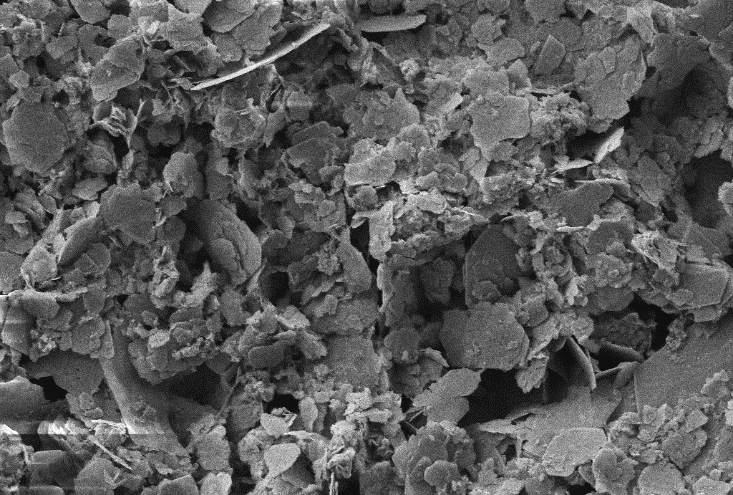


4 μm


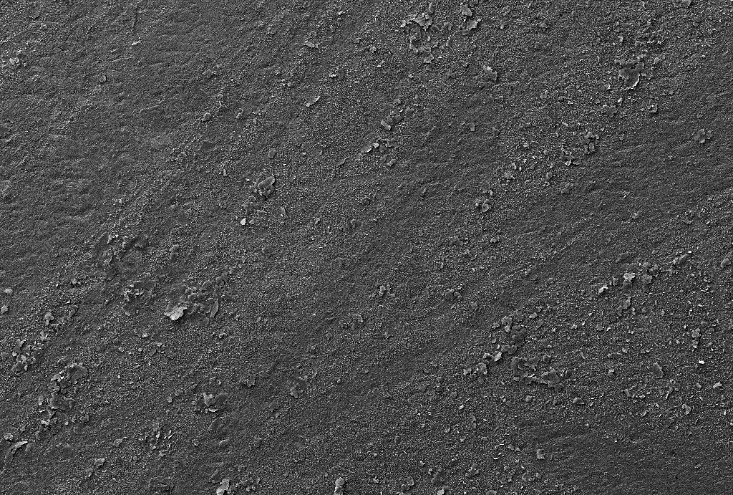

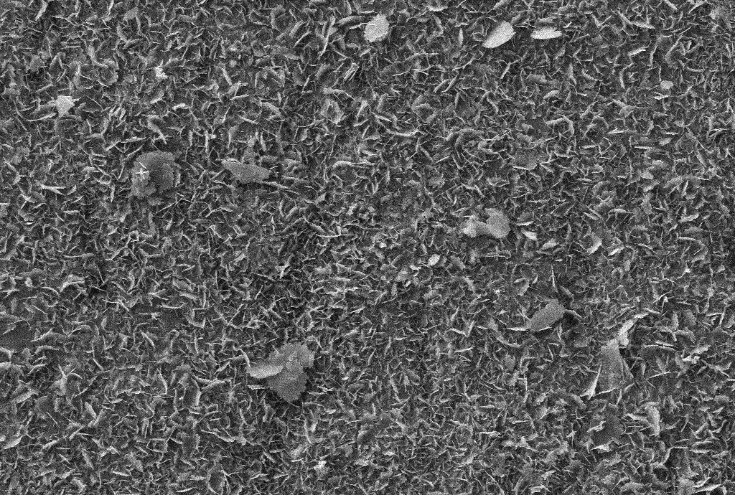


4 μm

80 μm

(a)

(b)

Zn-PVA

Zn-DHPVA

**Figure S11.** Initial cycles of Zn/Zn cells at 5 mA cm^-2^/5 mAh cm^-2^.

**Figure S12.** Characteristics of NaV_3_O_8_•1.5H_2_O cathode material. (a-b) SEM images. (c) XRD diffractograms.


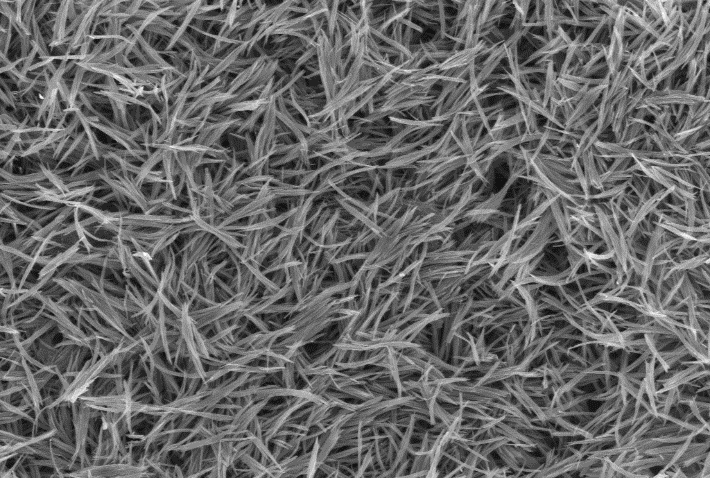


8 μm


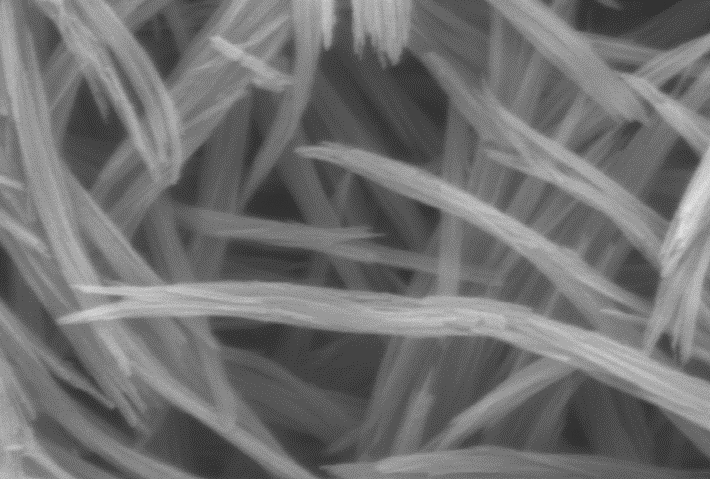


500 nm

(a)

(b)

(c)

**Figure S13.** CV plots of Zn/ NaV_3_O_8_ full cells at the different scan rates, (a) DHPVA cells, (b) PVA cells.

(a)

(b)

**Figure S14.** DRT profiles before cycling.


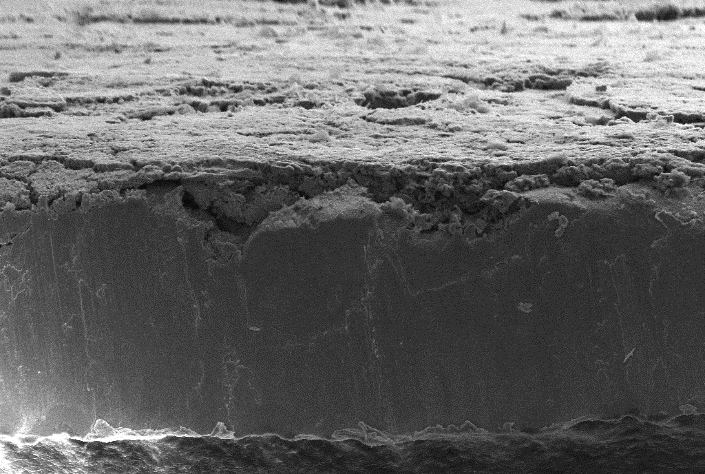


20 μm


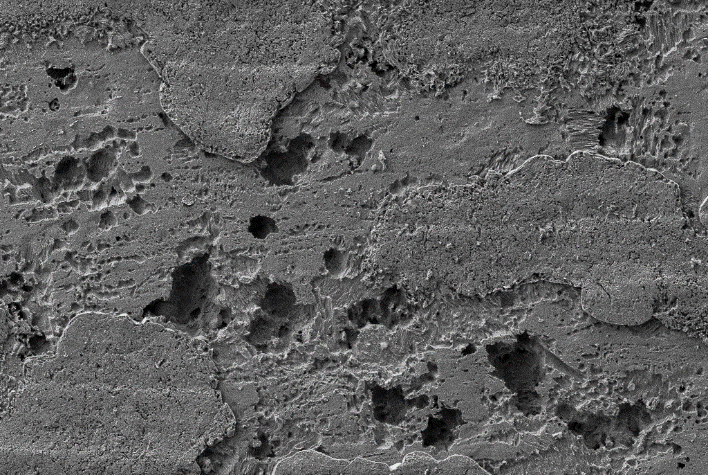


80 μm

PVA

**Loose dendrites layer**

Side-view


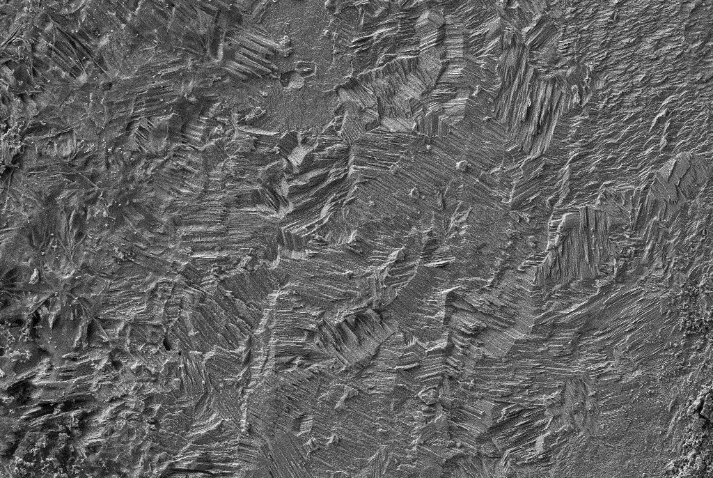


80 μm

DHPVA


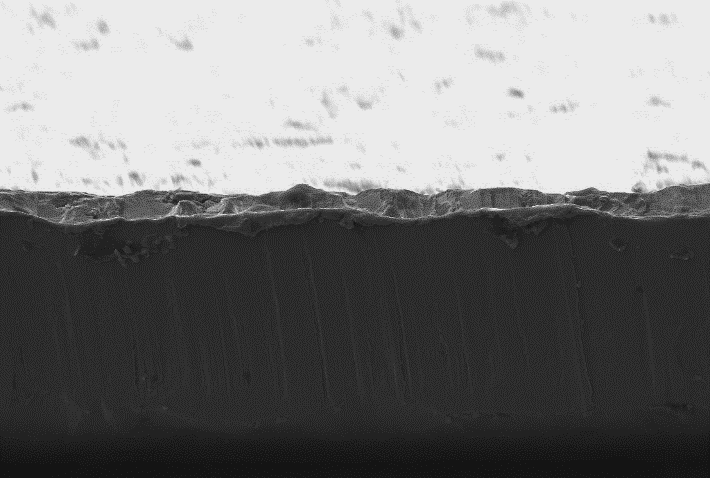


20 μm

**Dense layer**

Side-view

(a)

(b)

(c)

(d)

**Figure S15.** SEM images of Zn anodes after 100 cycles at 2 A g^-1^ in full cells with (a-b) DHPVA and (c-d) PVA.

**Figure S16.** The abnormal charge-discharge curve of PVA cell at 602^th^ cycle under 5 A g^-1^.

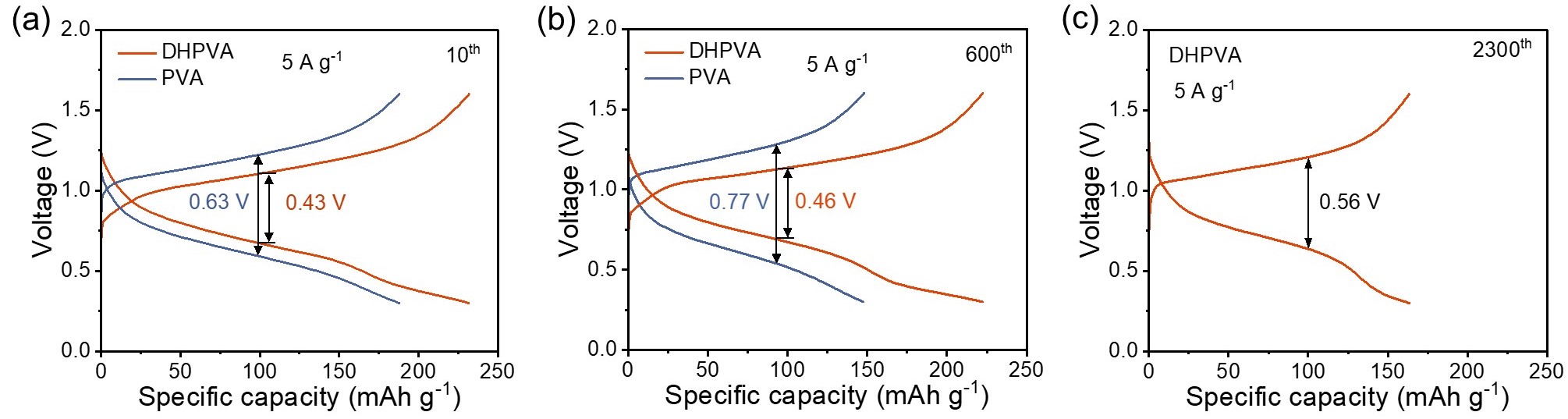


**Figure S17.** The charge-discharge curves of full cells with different separators at (a) 10^th^, (b) 600^th^ and (b) 2300^th^ cycle.


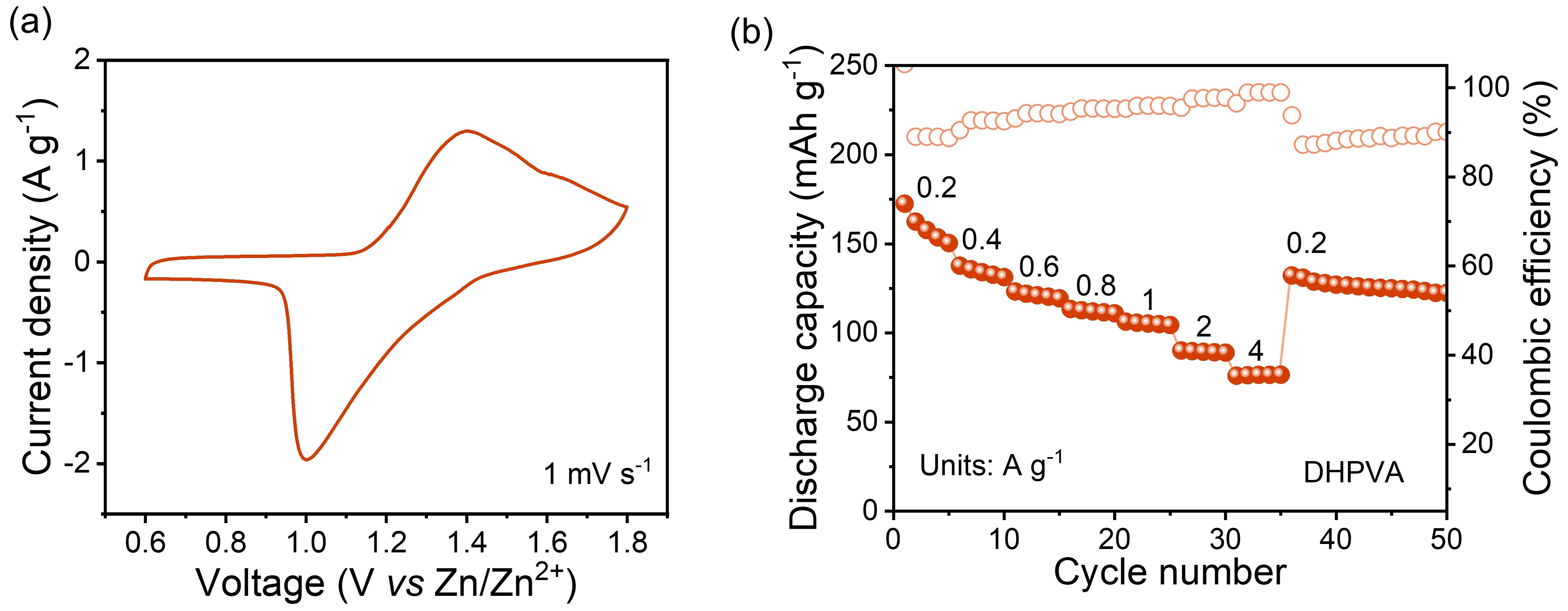


**Figure S18.** Electrochemical performance of a Zn/I_2_ full cell. (a) CV plot at a scan rate 1 mV s^-1^ with a voltage range from 0.6 to 1.8 V. (b) Rate capability performance.

Table S1. Ionic conductivity comparison of recently reported polymer-based separators for Zn-based batteries.

| Polymer-based separator | Ionic conductivity (mS cm^-1^) | Reference |
| --- | --- | --- |
| Polyacrylonitrile@graphene oxide | 7.69 | [6] |
| Polypropylene grafted acrylic acid | 3.16 | [7] |
| ZSM-5 molecular sieve | 3.7 | [8] |
| Carbonylation modification cellulose membrane | 1.04 | [9] |
| Polymer-matrixed zeolite | 11.3 | [2] |
| g-C_3_N_4_@cellulose fiber | 3.05 | [10] |
| PTFE | 23.8 | [11] |
| Cellulose nanofibers-ZrO_2_ | 4.59 | [12] |
| UiO-66-(COOH)_2_@GF | 15.1 | [13] |
| PVDF@GF | 11.2 | [14] |
| DHPVA | 12.5 | This work |

Table S2. Performance comparison of full cells with vanadium-based cathodes cycled at 5 A g^-1^

| Separator | Cathode | Electrolyte | Initial capacity (mAh g^-1^) at 5 A g^-1^ | Retention after 1000 cycles | Ref. |
| --- | --- | --- | --- | --- | --- |
| Graphene/GF | V_2_O_5_ | 2M ZnSO_4_ | 160 | 75% | [15] |
| PAN | NH_4_V_4_O_10_ | 2M ZnSO_4_ | 253 | 84% | [16] |
| PVDF@PDA | NH_4_V_4_O_10_ | 3M ZnSO_4_ | 208 | 92% | [17] |
| SPSF@PMIA | V_2_O_5_ | 2M Zn(CF_3_SO_3_)_2_ | 173 | 75% | [18] |
| Zn-MBA/non-woven fabrics | V_2_O_5_ | 3M Zn(CF_3_SO_3_)_2_ | 265 | 84% | [19] |
| N-doped carbon/GF | KV_12_O_30-y_ | 2M ZnSO_4_ | 251 | 79% | [20] |
| Polymer-matrixed zeolite | NaV_3_O_8_ | 1M Zn(CF_3_SO_3_)_2_ | 252 | 79% | [2] |
| DHPVA | NaV_3_O_8_ | 1M Zn(CF_3_SO_3_)_2_ | 224 | 88% | This work |

**References**

[1] F. Wan, L. Zhang, X. Dai, X. Wang, Z. Niu, J. Chen, *Nat. Commun.* **2018**, *9*, 1656.

[2] Y. Qin, X. Wang, *Angew. Chem. Int. Ed.* **2024**, *63*, e202315464.

[3] T. Wan, M. Saccoccio, C. Chena, F. Ciucci, *Electrochim. Acta* **2015**, *184*, 483-499.

[4] J. Wang, R. M. Wolf, J. W. Caldwell, P. A. Kollman, D. A. Case, *Comput. Chem.* **2004**, *25*, 1157-1174.

[5] A. W. Sousa da Silva, W. F. Vranken, *BMC Research Notes* **2012**, *5*, 367.

[6] L. Yao, C. Hou, M. Liu, H. Chen, Q. Zhao, Y. Zhao, Y. Wang, L. Liu, Z. Yin, J. Qiu, S. Li, R. Qin, F. Pan, *Adv. Funct. Mater.* **2022**, 2209301.

[7] X. Zhu, Z. Xu, T. Zhang, J. Zhang, Y. Guo, M. Shan, K. Wang, T. Shi, G. Cui, F. Wang, G. Xu, M. Zhu, *Adv. Funct. Mater.* **2024**, 2407262.

[8] J. Zhu, Z. Bie, X. Cai, Z. Jiao, Z. Wang, J. Tao, W. Song, H. Fan, *Adv. Mater.* **2022**, *34*, 2207209.

[9] Y. Zhang, Z. Liu, X. Li, L. Fan, Y. Shuai, N. Zhang, *Adv. Energy Mater.* **2023**, *13*, 2302126.

[10] Y. Yang, T. Chen, B. Yu, M. Zhu, F. Meng, W. Shi, M. Zhang, Z. Qi, K. Zeng, J. Xue, *Chem. Eng. J.* **2022**, *433*, 134077.

[11] G. Wu, R. Zhu, W. Yang, Y. Yang, J. Okagaki, Z. Lu, J. Sun, H. Yang, E. Yoo, *Adv. Funct. Mater.* **2024**, *34*, 2316619.

[12] J. Cao, D. Zhang, C. Gu, X. Zhang, M. Okhawilai, S. Wang, J. Han, J. Qin, Y. Huang, *Nano Energy* **2021**, *89*, 106322.

[13] P. Yang, K. Zhang, S. Liu, W. Zhuang, Z. Shao, K. Zhu, L. Lin, G. Guo, W. Wang, Q. Zhang, Y. Yao, *Adv. Funct. Mater.* **2024**, 2410712.

[14] F. Shen, H. Du, H. Qin, Z. Wei, W. Kuang, N. Hu, W. Lv, Z. Yi, D. Huang, Z. Chen, H. He, *Small* **2024**, *20*, 2305119.

[15] C. Li, Z. Sun, T. Yang, L. Yu, N. Wei, Z. Tian, J. Cai, J. Lv, Y. Shao, M. H. Rummeli, J. Sun, Z. Liu, *Adv. Mater.* **2020**, *32*, e2003425.

[16] Y. Fang, X. Xie, B. Zhang, Y. Chai, B. Lu, M. Liu, J. Zhou, S. Liang, *Adv. Funct. Mater.* **2021**, *32*, 2109671.

[17] Y. Liu, S. Liu, X. Xie, Z. Li, P. Wang, B. Lu, S. Liang, Y. Tang, J. Zhou, *InfoMat* **2022**, *5*, e12374.

[18] W. Hu, J. Ju, Y. Zhang, W. Tan, N. Deng, W. Liu, W. Kang, B. Cheng, *J. Mater. Chem. A* **2022**, *10*, 24761-24771.

[19] Z. Tao, J. Cui, Y. Tan, Z. Zhou, Z. Chen, A. Wang, Y. Zhu, S. Lai, M. Yu, Y. Yang, *Small* **2023**, e2301620.

[20] X. Yang, W. Li, J. Lv, G. Sun, Z. Shi, Y. Su, X. Lian, Y. Shao, A. Zhi, X. Tian, X. Bai, Z. Liu, J. Sun, *Nano Research* **2021**, *15*, 9785-9791.
